# Post-Traumatic Stress Disorder Is Associated with Elevated Plasma Cholesterol in Female TT Homozygotes of *LDLR* rs5925

**DOI:** 10.3390/ijms24109016

**Published:** 2023-05-19

**Authors:** Jinhua Wang, Kexin Jia, Qiwei Guo, Junyi Liu, Jiajing Cai, Yilin Shen, Guoming Su, Xu Chen, Jia Lin, Dingzhi Fang

**Affiliations:** Department of Biochemistry and Molecular Biology, West China School of Basic Medical Sciences & Forensic Medicine, Sichuan University, Chengdu 610041, China

**Keywords:** adolescents, posttraumatic stress disorder, low-density lipoprotein receptor, serum lipids, *LDLR* rs5925

## Abstract

To explore the mechanism of inconsistent relationships between plasma lipid profiles and post-traumatic stress disorder (PTSD) reported before, we hypothesized that interplays might exist between PTSD and a variation of rs5925 at low-density lipoprotein receptor (*LDLR*) gene on plasma lipid profiles. To test our hypothesis, we analyzed the plasma lipid profiles of 709 high school pupils with various genotypes of *LDLR* rs5925 and with or without PTSD. The results demonstrated that PTSD prevalence in the C allele carriers was higher than that in the TT homozygotes regardless of gender. The C allele carriers had higher levels of total cholesterol (TC), low-density lipoprotein cholesterol (LDL-C), ratios of TC to high-density lipoprotein cholesterol (TC/HDL-C) and LDL-C/HDL-C than the TT homozygotes in the male controls, and only higher TC in the female controls, but no differences in the male or female PTSD subjects. PTSD increased TC in the female TT homozygotes but not in the female C allele carriers. PTSD increased TC/HDL-C in the male TT homozygotes but not in the C allele carriers. These results suggest interactions between PTSD and *LDLR* rs5925 on plasma lipid profiles, which may be among the explanations for previously reported inconsistent relationships between *LDLR* rs5925 or PTSD and plasma lipid profiles, and facilitate the development of precision medicine interferences in hypercholesterolemia in individuals with different genetic backgrounds and psychiatric status. Psychiatric care or drug supplement may particularly be needed by female hypercholesterolemic subjects with the TT genotype of *LDLR* rs5925 in Chinese adolescents.

## 1. Introduction

Post-traumatic stress disorder (PTSD) is a psychiatric disorder [1,2] and has been observed to be associated with somatic disturbances [3]. Previous reports have indicated that PTSD is correlated with the development of cardiovascular disease (CVD) [4,5,6]. Meanwhile, researchers proved that patients with PTSD maintained substantially lower levels of high-density lipoprotein cholesterol (HDL-C) and higher levels of total cholesterol (TC), triglyceride (TG), and low-density lipoprotein cholesterol (LDL-C) than the control group. [7,8]. However, no significant differences of serum LDL-C levels were observed by some other studies between participants with and without PTSD [9]. More interestingly, it was also reported that there were significantly lower LDL-C levels in male patients with PTSD [10]. The mechanism of the controversial correlation between serum lipid profiles and PTSD has not been elucidated yet. Although genetic characteristics were observed to be associated with serum lipid profiles as well as PTSD, more studies are needed to explore their interplays on serum lipid profiles.

Low-density lipoprotein receptor (LDLR), a surface receptor in the cytoplasm membrane, plays a role in removing LDL-C from plasma and maintaining cholesterol homeostasis. The gene encoding LDLR (*LDLR*) is situated on the short arm of chromosome 19 [11,12]. Individuals with mutations of *LDLR* showed different levels of LDL cholesterol, risk of familial hypercholesterolemia and frequencies of CVD [13,14,15]. Some recent studies showed that the polymorphism of *LDLR* rs5925 was a common contributing factor to the changes in serum lipid levels in Chinese populations [16]. Individuals carrying the C allele presented higher serum levels of LDL-C, TC, and TG compared to the TT homozygotes [17]. Meanwhile, the frequency of the *LDLR* rs5925 variant differs between the Chinese population and others [18,19,20]. Furthermore, studies have revealed that *LDLR* is also related to mood disorders. Studies on *LDLR* (−/−) mice demonstrated that *LDLR* was involved in developing depressive-like behavior [21]. Our previous research demonstrated a timing-dependent association between *LDLR* rs5925 and the frequency and severeness of PTSD in teenagers after the Wenchuan earthquake. [22]. However, more attempts are needed to explore the relationship between *LDLR* mutations and PTSD in human beings, including the relationship between *LDLR* rs5925 and PTSD.

Therefore, to explore the possible mechanism of the controversial relationships reported before between plasma lipid profiles and PTSD, and further explore the factors affecting plasma lipid profiles, we hypothesized that there might be interplays between PTSD and *LDLR* variations in male and female subjects on the plasma lipid profiles. To test our hypothesis in the present investigation, plasma lipid profiles of high school students who had distinct genotypes of *LDLR* rs5925 and with or without PTSD were examined. Since studies demonstrated that the ratios of plasma lipid levels were associated with CVD and had better predictive value than the conventional lipid levels [23], lipid ratios of TG/HDL-C, LDL-C/HDL-C, and TC/HDL-C were also examined in the present study.

## 2. Results

### 2.1. LDLR rs5925 Genotype and Frequency of Alleles

*LDLR* rs5925 genotype and frequency of alleles in the subjects of the current study are presented in Table 1. The genotype frequency distribution was in accordance with Hardy–Weinberg equilibrium. No significant differences between the male and female subjects were observed in the genotype and allele frequencies of *LDLR* rs5925. Due to a limited number of subjects with the CC genotype, they were combined with the CT heterozygotes and depicted as C allele carriers (including CC/CT) in the tables and for subsequent analyses.

### 2.2. Prevalence of PTSD in Subjects with Different LDLR rs5925 Genotypes

To test the association of PTSD with *LDLR* rs5925, the prevalence of PTSD was examined in the subjects with different genotypes of *LDLR* rs5925 (Table 2). The results show that PTSD prevalence in the C allele carriers was higher than that in the TT homozygotes regardless of gender. Moreover, the female subjects had higher PTSD prevalence than the male subjects in the C allele carriers, but not in the TT homozygotes.

### 2.3. Anthropometric Characteristics and Plasma Lipid Profiles of the Subjects with Different Genotypes of LDLR rs5925

**Figure 1:**
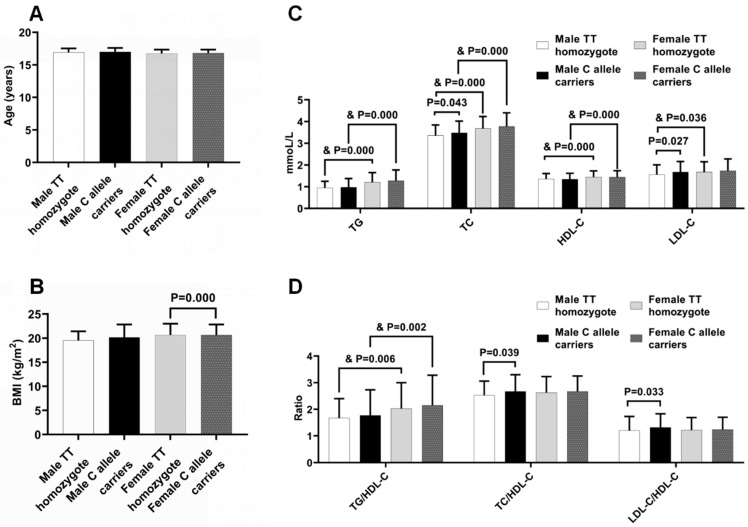
Anthropometric characteristics and plasma lipid profiles in subjects with different genotypes of *LDLR* rs5925. (**A**) Age. (**B**) BMI. (**C**) TG, TC, HDL-C, LDL-C. (**D**) TG/HDL-C, TC/HDL-C, LDL-C/HDL-C. Body mass index (BMI), triglycerides (TG), total cholesterol (TC), HDL-C, and low-density lipoprotein (LDL-C) cholesterol. ^&^ *p* values, when compared with those of males after the adjustment for BMI (Analyses of covariance). The remaining *p* values, when compared with those of males (One-way analyses of variance), or when compared to those of TT homozygotes (One-way analyses of variance).

To test the association of plasma lipid profiles with *LDLR* rs5925, the levels of plasma lipids and their ratios were analyzed in the subjects with different genotypes of *LDLR* rs5925 (Figure 1). Since gender is an important confounding factor affecting serum lipid profiles, the analyses were performed separately on male and female subjects. Regardless of genotype, there were no significant differences in age between the male and female pupils, but the female subjects had a higher BMI than their male counterparts. Therefore, the following comparisons between the male and female subjects were adjusted by BMI. There were higher levels of TG, TC, HDL-C, and TG/HDL-C in the female students than those in the male subjects regardless of the genotypes, and higher levels of LDL-C only in the TT homozygotes, but not in the C allele carriers, after the adjustment of BMI. Meanwhile, there were no significant differences of age, BMI, TG, HDL-C, and TG/HDL-C between the TT homozygotes and the C allele carriers regardless of gender. Nevertheless, the C allele carriers had higher levels of TC, LDL-C, TC/HDL-C, and LDL-C/HDL-C than the TT homozygotes in male subjects (Figure 1), but not in female subjects.

### 2.4. Anthropometric Characteristics and Plasma Lipid Profiles in the Subjects with Different Genotypes of LDLR rs5925 and with or without PTSD

**Figure 2:**
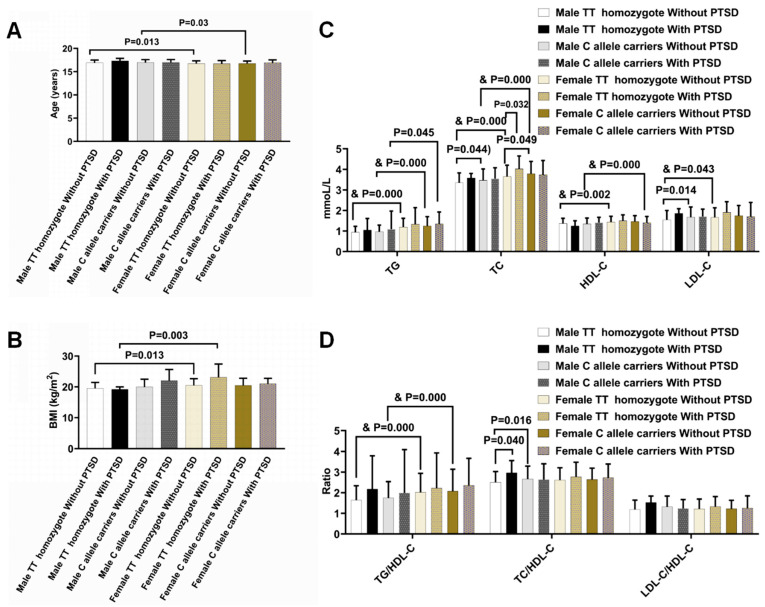
Anthropometric characteristics and plasma lipid profiles in the subjects with different genotypes of *LDLR* rs5925 and with or without PTSD. (**A**) Age. (**B**) BMI. (**C**) TG, TC, HDL-C, LDL-C. (**D**) TG/HDL-C, TC/HDL-C, LDL-C/HDL-C. Body mass index (BMI), triglycerides (TG), total cholesterol (TC), HDL-C, and low-density lipoprotein (LDL-C) cholesterol. ^&^ *p* values, when compared with those of males after the adjustment for BMI and/or age (Analyses of covariance). The remaining *p* values, when compared with those of subjects without PTSD (One-way analyses of variance), or when compared with those of males (One-way analyses of variance), or when compared with those of TT genotype (One-way analyses of variance).

To explore the interplays of PTSD with *LDLR* rs5925 on plasma lipid profiles, anthropometric characteristics and plasma lipid profiles were investigated in the subjects with different genotypes of *LDLR* rs5925 and with or without PTSD (Figure 2). The analyses were adjusted by age and/or BMI once they were significantly different because they were important confounding factors affecting plasma lipid profiles. When tested between the subjects with different genotypes, no significant differences were observed in the male or female PTSD subjects. Nevertheless, the C allele carriers had significantly higher levels of TC, LDL-C, TC/HDL-C, and LDL-C/HDL-C than the TT homozygotes in the male controls, but only higher levels of TC in the female controls. When we tested between subjects with different genders, the female control subjects had higher levels of TG, TC, HDL-C, LDL-C, and TG/HDL-C than their male control counterparts after adjustment for age and BMI in the TT homozygotes, and higher levels of TG, TC, HDL-C, and TG/HDL-C after adjustment for age in the C allele carriers. There were higher levels of TG in the female PTSD subjects than their male PTSD counterparts in the C allele carriers, but no significant differences between the female PTSD subjects and their male PTSD counterparts before and after the adjustment of BMI in the TT homozygotes. When testing between the subjects with and without PTSD, the subjects with PTSD had higher levels of TC than the controls in the female TT homozygotes and TC/HDL-C in the male TT homozygotes. No significant differences were found between the subjects with and without PTSD in the C allele carriers irrespective of gender (Figure 2).

## 3. Discussion

The relationship between PTSD and plasma lipid levels has been intensively investigated [24]. Because *LDLR* variations were the key players in the regulation of plasma lipid levels [25], more efforts are needed to test the relationship between PTSD and *LDLR* variations and their interplays on plasma lipid profiles. Epidemiological studies have reported the latter to be associated with mood disorders [26]. In fact, depressive-like behaviors were reported in *LDLR* (−/−) mice by the reduction in the grooming time in splash tests, increased immobility time in forced swimming tests, and increased activity of monoamine oxidase A and decreased hemeoxygenase-1 mRNA levels in the hippocampus [21]. This finding was confirmed by sucrose preference tests, splash tests, and tail suspension tests, as well as elevated monoamine oxidase A and B reactivity in the hippocampus of *LDLR* (−/−) mice. [27]. In the present study, *LDLR* rs5925 was selected because it was a common contributing factor to the changes in serum lipid levels in Chinese populations. Meanwhile, the current study was carried out in adolescents because they were more easily affected by traumatic stress such as earthquake to have PTSD [28,29,30] and their prevalence of hyperlipidemia was steadily increased [31]. The frequencies of TT, TC, and CC genotypes were observed to be higher, lower, and lower, respectively, and similar to the results reported by others [32]. The female subjects were found to have higher PTSD prevalence than the male counterparts only in the C allele carriers, but not in the TT homozygotes of *LDLR* rs5925. Moreover, PTSD prevalence in the C allele carriers was observed to be higher than that in the TT homozygotes regardless of gender (Table 2). The results demonstrate that *LDLR* rs5925 is associated with PTSD.

*LDLR* rs5925 was demonstrated to be associated with plasma lipid profiles [33]. The individuals carrying the C allele of *LDLR* rs5925 were observed to have higher levels of TC, TG, and LDL-C when compared to the TT homozygotes in some investigations [17,18], although only higher levels of TG in another study [34]. On the other hand, this variant was also reported to be associated with lower levels of plasma LDL-C in Italian individuals [35]. However, *LDLR* rs5925 was not associated with the lipid profile change in European subjects from Germany, the Netherlands, and Denmark [36]. According to the findings in Amerindian Chilean participants, there were no differences in plasma levels of TC, TG, and LDL-C between the subjects with the wild genetic type and the subjects with the variant of *LDLR* rs5925 [37]. Although ethnic characteristics in the studied individuals were hypothesized to be one of the factors for the above discrepancies, the mechanism of the inconsistent relationship between *LDLR* rs5925 and plasma lipid profiles has not been elucidated yet. In addition, similar inconsistent relationships between PTSD and plasma lipid profiles were also reported before. In the present study, the C allele carriers were found to have higher levels of TC, LDL-C, TC/HDL-C, and LDL-C/HDL-C than the TT homozygotes in the male controls, and only higher levels of TC in the female controls, but no differences in the male or female PTSD subjects. Furthermore, the female control subjects were observed to have higher levels of TG, TC, HDL-C, LDL-C, and TG/HDL-C than their male control counterparts in the TT homozygotes, and higher levels of TG, TC, HDL-C and TG/HDL-C in the C allele carriers, while the female PTSD subjects had higher levels of TG than their male PTSD counterparts in C allele carriers, but there were no differences in their TT homozygotes. Moreover, the PTSD subjects had higher levels of TC than the controls in the female TT homozygotes and TC/HDL-C in the male TT homozygotes, but no differences were found in the C allele carriers irrespective of gender (Figure 2). These results suggest that there were interplays between *LDLR* rs5925 and PTSD to influence the serum lipid levels and their ratios in a gender-dependent manner.

The *LDLR* rs5925 has been confirmed to be a functional single-nucleotide polymorphism. It has been observed that empirical programs can modify exon-splicing enhancers in silico. This polymorphism variation is sufficient to explain the differences of LDLR involving exon 13 in splicing efficiency. The C allele can result in enhanced exon 13 splicing efficacy in *LDLR* [38]. Therefore, the effects *LDLR* rs5925 observed in the present study on the prevalence of PTSD and plasma lipid profiles in the male and female subjects with or without PTSD may be due to the difference of splicing efficiency. However, other mechanisms such as linkage disequilibrium, haplotype block, and its interactions with other factors cannot be excluded because PTSD and plasma lipid profiles are typical complex characteristics affected by a series of factors [39,40,41,42]. For example, sex hormones can be among the factors because the results of the present study indicate that interplays between *LDLR* rs5925 and PTSD regulate the levels of serum lipid levels and their ratios in a gender-dependent manner.

There were numerous limitations to this investigation. First, LDLR mRNA and protein levels were not measured. However, there are still benefits to analyze the associations without testing mRNAs and proteins, as the differences of mRNAs and proteins are not the only mechanisms underlying the associations between genetic mutations and phenotypes; linkage disequilibrium and haplotype block can also be involved. Secondly, PTSD was defined only by the PTSD Checklist—Civilian Version. These restrictions should be considered while explaining this study’s outcomes.

## 4. Materials and Methods

### 4.1. Study Population

This study was approved by the Human Ethics Committee of Sichuan University. Written consent was obtained from all the participants and their guardians. The participants were chosen from a high school 10 km away from the epicenter of the 2008 Wenchuan earthquake, 6 months after the earthquake. Although no building collapsed, all the buildings at the school were more or less damaged, and all the participants studied and lived in shelters during the present investigation. The recruitment criteria included comprehension of the study’s procedures, absence of a metabolic disease history, and provision of blood samples. Volunteers with cardiovascular, kidney, or endocrinological disorders, those taking lipid-lowering medications or hormones, and those who drank alcohol or smoked were excluded from the study. The study included a total of 709 participants, all of whom were Chinese Han.

### 4.2. Questionnaires and Measurements

The symptoms of PTSD were assessed using the PTSD Checklist—Civilian Version (PCL-C) [43], which was based on the 4th edition (DSM-IV) criteria. This measurement had been widely used in adolescents and been proven to have high internal consistency [44,45]. It is a 17-item self-report scale with total scores from 17 to 85. A total score of 50 was set as the cutoff point of PTSD diagnosis [46] in the present study.

The levels of plasma TC, TG, HDL-C, and LDL-C were measured using usual methods. Briefly, TC and TG were quantified enzymatically with the aid of a semi-automated biochemistry analyzer (Cobas 6000, F. Hoffmann-La Roche, Ltd., Mannheim, Germany). LDL-C was measured using polyvinyl sulfate precipitation and enzymatic testing on a semi-automated biochemistry analyzer(Cobas 6000, F. Hoffmann-La Roche, Ltd., Germany). After precipitating apolipoprotein B-containing lipoproteins with phos-photungstic-Mg^2+^, HDL-C was measured enzymatically. All the plasma was measured 3 times, and the average was used for statistical analyses. Then the average was used to calculate TG/HDL-C, TC/HDL-C, and LDL-C/HDL-C.

### 4.3. DNA Extraction and Genotyping

The peripheral leukocytes’ genomic DNA was extracted using a commercial DNA extraction reagent (Beijing Tian Enze Gene Technology Co., Beijing, China) and stored at −80 °C. All of the aforementioned examinations were conducted six months after the 2008 earthquake. The tests and examinations listed below were conducted in 2018. *LDLR* rs5925 was genotyped using polymerase chain reaction and restriction fragment length polymorphism (PCR-RFLP) and confirmed by DNA sequencing (Sangon Biotech (Shanghai) Co., Shanghai, China). A 228 bp sequence containing *LDLR* rs5925 was amplified by PCR with the forward primer 5′-GTCATCTTCCTTGCTGCCTGTTTAG-3′ and reverse primer 5′-GTTTCCACAAGGAGGTTTCAAGGTT-3′ [17] (Sangon Biotech (Shanghai) Co., China). After denaturation at 94 °C for 3 min, 35 cycles of denaturation at 94 °C for 30 s, annealing at 56 °C for 30 s, and 72 °C extending for 60 s were conducted, followed by a final extension of 5 min at 72 °C. The PCR products were digested overnight at 37 °C with the *Ava* II enzyme. By 3% agarose gel electrophoresis, the TT genotype of *LDLR* rs5925 fragments migrated as a 228 bp band, the CC genotype as a 134 bp and a 94 bp band, and the CT genotype as a 134 bp, a 94 bp, and a 228 bp band. These bands were visualized using ultraviolet illumination with Gold-view staining.

### 4.4. Statistical Analyses

The results are expressed in terms of means and standard deviations (S.D.) unless otherwise stated. Statistical significance was considered as *p* ≤ 0.05. The goodness-of-fit test was performed to examine the agreement of *LDLR* rs5925 genotype distribution with the Hardy–Weinberg equilibrium. Chi-square tests were utilized to examine the disparities in the genotypes of *LDLR* rs5925 and the frequency of PTSD between males and females, as well as the frequency of PTSD between subjects with distinct genotypes of *LDLR* rs5925. One-way analyses of variance (ANOVA) were utilized to analyze the differences of anthropometric characteristics and plasma lipid profiles in the subjects with different genders, different genotypes of *LDLR* rs5925, or with or without PTSD. Analyses of covariance (ANCOVA) were performed to compare the differences of plasma lipid profiles in the subjects with different genders, different genotypes of *LDLR* rs5925, or with or without PTSD using age or/and Body Mass Index (BMI) as a covariate or covariates when age or/and BMI was/were significantly different because the impacts of these factors were observed on plasma lipid levels.

## 5. Conclusions

In conclusion, the current study demonstrated that interplays existed between *LDLR* rs5925 and PTSD in a gender-dependent manner. PTSD was associated with increased levels of plasma TC in the female TT homozygotes, but not in the female C allele carriers. In the male subjects, PTSD was linked to elevated levels of TC/HDL-C in the TT homozygotes, but not in the C allele carriers, suggesting some similar effects of PTSD on the plasma cholesterol levels as in the female subjects. This finding may be one of the explanations for previously reported inconsistencies between *LDLR* rs5925 or PTSD and plasma lipid profiles, and make it possible for precise medical interferences of hypercholesterolemia among subjects with distinct genetic backgrounds and psychiatric status. Therefore, psychiatric care or drug supplement may particularly be needed by the female hypercholesterolemic subjects with the TT genotype of *LDLR* rs5925 in the Chinese population.

## Figures and Tables

**Table 1:** Frequencies of *LDLR* rs5925 alleles and genotypes in the study population.

	Total (n = 709) *n* (%)	Hardy–Weinberg *p*	Males (n = 312) *n* (%)	Females (n = 397) *n* (%)	*p*
Genotype frequencies					
TT	392 (55.29)	0.33	177 (56.73)	215 (54.16)	0.63
CT	277 (39.07)		116 (37.18)	161 (40.55)	
CC	40 (5.64)		19 (6.09)	21 (5.29)	
Allele frequencies					
T	1061 (74.82)		470 (75.32)	591 (74.43)	0.71
C	357 (25.18)		154 (24.68)	203 (25.57)	

**Table 2:** Prevalence of PTSD in the subjects with different genotypes of *LDLR* rs5925.

PTSD	TT Homozygote			C Allele Carriers				
	Males *n* (%)	Females *n* (%)	χ*^2^*, *p*	Males *n* (%)	Females *n* (%)	χ*^2^*, *p*	χ*^2^*, *p*	χ*^2^*, *p*
With	6 (3.39)	11 (5.12)	χ^2^ = 0.697,	12 (8.89)	46 (25.27)	χ^2^ = 13.921,	χ^2^ = 4.260,	χ^2^ = 32.573,
Without	171 (96.61)	204 (94.88)	*p* = 0.403	123 (91.11)	136 (74.73)	*p* = 0.000	* *p* = 0.039	# *p* = 0.000

Data are expressed as n (%). * Comparison of different genotypes in males (Chi-Square test). # Comparison of different genotypes in females (Chi-Square test).

## Data Availability

Derived data supporting the findings of this study are available from the corresponding author upon reasonable request.

## Abbreviation

PTSD	Post-traumatic stress disorder
LDLR	Low-density lipoprotein receptor
TC	Total cholesterol
LDL-C	Low-density lipoprotein cholesterol
HDL-C	High-density lipoprotein cholesterol
TG	Triglyceride
PCL-C	PTSD Checklist—Civilian Version
BMI	Body Mass Index
